# Prevention at home in older persons with (pre-)frailty: analysis of participants’ recruitment and characteristics of the randomized controlled PromeTheus trial

**DOI:** 10.1007/s40520-024-02775-x

**Published:** 2024-05-23

**Authors:** Tim Fleiner, Corinna Nerz, Michael Denkinger, Jürgen M. Bauer, Christian Grüneberg, Judith Dams, Martina Schäufele, Gisela Büchele, PromeTheus Study Group, Kilian Rapp, Christian Werner

**Affiliations:** 1 Institute for Geriatric Research, Ulm University Medical Center, Ulm, Germany; 2 Geriatric Center, Agaplesion Bethesda Clinic, Ulm, Germany; 3 Department of Clinical Gerontology, Robert-Bosch-Hospital, Stuttgart, Germany; 4 Geriatric Center, Heidelberg University Hospital, Agaplesion Bethanien Hospital Heidelberg, Heidelberg, Germany; 5 Department of Applied Health Sciences, Hochschule für Gesundheit (University of Applied Sciences), Bochum, Germany; 6 Department of Health Economics and Health Services Research, University Medical Centre Hamburg-Eppendorf, Hamburg, Germany; 7 Department of Social Work, University of Applied Sciences, Mannheim, Germany; 8 Institute of Epidemiology and Medical Biometry, Ulm University, Ulm, Germany

**Keywords:** (pre)frailty, Community-dwelling, Prevention, Recruitment

## Abstract

**Background:**

The “PromeTheus” trial is evaluating a home-based, multifactorial, interdisciplinary prevention program for community-dwelling (pre-)frail older adults. These individuals often suffer from reduced participation, which can complicate the recruitment and enrollment in a clinical trial.

**Aims:**

The aim of this study was to evaluate different recruitment strategies and differences in participant characteristics in relation to these strategies.

**Methods:**

This cross-sectional study used baseline data from the randomized-controlled PromeTheus trial, in which community-dwelling (pre-)frail older persons (Clinical Frailty Scale [CFS] 4–6 pt., ≥ 70 years) were recruited via general practitioners (“GP recruitment”) or flyers, newspaper articles, and personalized letters (“direct recruitment”). Differences in the sociodemographic, clinical, physical, functional, mobility-related, psychological and social characteristics were analyzed in relation to the recruitment strategy.

**Results:**

A total of 385 participants (mean age = 81.2, SD 5.9 years; women: *n* = 283, 73.5%) were enrolled, of which 60 (16%) were recruited by GPs and 325 (84%) through direct recruitment. Participants recruited via GPs had significantly higher subjective frailty levels (CFS), were more often physically frail (Fried Frailty Phenotype), and showed lower physical capacity (Short Physical Performance Battery), participation (disability component of the short version of the Late-Life Function and Disability Instrument), and life-space mobility (Life-Space Assessment) compared to those recruited via the direct approach (*p* = 0.002–0.026). Costs per randomized participant were 94€ for the GP recruitment strategy and €213 for the direct recruitment strategy.

**Conclusion:**

Different strategies may be required to successfully recruit (pre-)frail home-living older adults into preventive programs. Direct recruitment strategies, in which potential participants are directly informed about the prevention program, seem to be more promising than GP recruitment but may result in enrolment of persons with less functional impairment and higher recruitment costs.

**Trial registration:**

German Clinical Trials Register, DRKS00024638. Registered on March 11, 2021.

## Background

Among community-dwelling adults aged 70 years and older, the prevalence of frailty is estimated to be 20–31% and pre-frailty about 50%, depending on the frailty instrument used (e.g., Fried Frailty Phenotype [FFP], Frailty Index) [1]. Frailty is characterized by a decline in physiological reserves and resistance to stressors leading to increased vulnerability to adverse health outcomes, a higher risk of decompensation for home care, and greater healthcare utilization and costs [2, 3]. Implementing preventive measures for (pre-)frail older people is a key challenge for healthcare systems in aging societies, and effective strategies to prevent or reduce physical decline and frailty are deliberately needed to enable them to live socially integrated and independently at home for as long as possible [4, 5].

Currently, outpatient physiotherapy or inpatient geriatric rehabilitation are the health services prescribed by general practitioners (GPs) in Germany to counteract the steady decline in physical capacity and daily functioning of community-dwelling (pre-)frail older people. However, common physiotherapy programs are usually only episodic, one-dimensional, not intensive, and not frequent enough and not sustainable. In addition, many older adults prefer to age in place, which is also more desirable from a health economic perspective, as it avoids the high costs for institutionalization [6]. However, other community-based alternatives to physiotherapy to prevent or reduce physical decline and frailty are not yet established. As part of the Australian Frailty Intervention Trial (FIT) project [7], a multifactorial, interdisciplinary intervention program was shown to be (cost-)effective in reducing frailty, mobility limitations, and the fall risk [8–10]. The main component of the FIT project is a home-based training program (WEBB = “Weight-bearing Exercise for Better Balance”) supervised by a physiotherapist, which can be complemented by optional counseling services [11]. The FIT intervention serves as a model for the PromeTheus project in Germany, which aims to establish a 12-month community-based prevention program for (pre-)frail older adults living at home [12].

Inclusion of (pre-)frail older people in clinical trials can be particularly challenging [13], and some researchers report of concerns about trial participation of this target group [14–17]. In contrast to this, older adults have been reported to be curious and interested in research that focuses on their needs and resources such as prevention, recognition, and management of frailty for research in later life [18, 19]. Confirming this high interest in prevention trials, previous recruitment analyses have reported that frailty was not associated with higher or lower trial participation rates [20]. Current evidence on the recruitment of (pre-)frail older adults in clinical trials points to the following three key aspects: (a) continuous monitoring of the recruitment strategy to adapt methods and study procedures, (b) the involvement of GPs and healthcare providers who know the people and can encourage them to participate (“GP recruitment”) (c) the use of newspaper advertisements, direct mailings, and personalized letters to approach potential participants directly (“direct recruitment”) [18, 21]. Effective recruitment of community-dwelling (pre-)frail older adults would also provide the basis for studying and establishing the FIT intervention as a preventive role model in German healthcare. However, there is a lack of knowledge about the effectiveness of different strategies to engage this population in preventive intervention programs.

Therefore, this analysis of the PromeTheus study aims to (a) evaluate the effectiveness of two different recruitment strategies (“GP recruitment” vs. “direct recruitment”) and (b) explore differences in participant characteristics in relation to the recruitment strategy.

## Methods

### Study design

This is a secondary analysis of baseline data of the PromeTheus study, a multicenter, assessor-blinded, randomized, controlled trial conducted in southern Germany with study sites in Stuttgart, Heidelberg, and Ulm. The PromeTheus study aims to analyze the effects of a 12-month multifactorial, interdisciplinary intervention on the prevention of functional and mobility decline in 400 community-dwelling (pre-)frail older adults. The detailed study protocol has been published elsewhere [12]. The PromeTheus study was approved by the local ethics committees at each study site (approval numbers: Stuttgart, #732/2020B01; Heidelberg, #S-072/2021; Ulm, #26/21) and the Ethics Committee of the State Medical Association of Baden-Wurttemberg (#B-F-2021-042). The PromeTheus was registered in the German Clinical Trials Register (DRKS00024638) on March 11, 2021.

### Recruitment and eligibility criteria

Recruitment of participants started in May 2021, with the goal of being carried out over the following 12 months by GPs registered with the study, ending at the end of April 2022. This process, referred to as “GP recruitment”, involved GPs identifying and preliminarily screening patients who met the eligibility criteria during routine visits. Eligible patients were those aged 70 years or older, living independently or in assisted living facilities, capable of walking ≥ 10 m with or without the aid of a walking device, pre-frail (Clinical Frailty Scale [CFS] = 4 pt.) or mildly (CFS = 5 pt.) or moderately (CFS = 6 pt.) frail [22–24], no medical contraindications (heart failure [NYHA III-IV], stroke within the last 6 months, Morbus Parkinson (Hoehn & Yahr Stage ≥ 3), advanced cancer, severe lung disease, multiple sclerosis), and members of the Allgemeine Ortskrankenkasse (AOK). The AOK is one of the largest providers of statutory health insurance in Germany, with approximately 27 million insured persons and a market share of more than one-third (37%) of all statutory health insurance members [25]. If eligibility was confirmed by the GP, the potential participant was referred to the study center. For each referral of a potential participant, the study-registered GPs received financial compensation via a supplementary contract. The study center then confirmed further exclusion criteria by a telephone screening (walking ability > 800 m without walking aid or breaks, inadequate German language skills/ visual acuity) and by an initial home visit (cognitive impairment = Short Orientation-Memory-Concentration Test [SOMCT] score > 10 pt. [26, 27]). If all eligibility criteria were met, the home visit was continued with the assessment measures.

During the COVID-19 pandemic German GPs were responsible for administering the COVID-19 vaccine in Germany [28] and had very limited capacity to screen or refer potential participants in 2021. Therefore, an alternative recruitment strategy focusing on direct communication (“direct recruitment”) was implemented and the recruitment period was extended to October 2022. Potential participants were informed in February 2021 and 2022 via flyers (*n* = 32,500 each) inserted in the local health magazines of the AOK health insurance company and via articles in local magazines and newspapers. In addition, personalized letters with study information and an eligibility assessment template for GPs were mailed to AOK members aged ≥ 70 years living at home or in assisted living facilities in the three study regions. A total of 33,796 letters were mailed to potential participants. Interested individuals contacted the local study center, were informed about the study, and were referred to a GP for eligibility assessment. The subsequent steps in the recruitment (telephone screening, initial home visit) were the same as for the GP recruitment strategy. The same inclusion and exclusion criteria were used for the GP recruitment strategy as well as for the direct recruitment strategy.

### Measurements

All data were collected in-person at participants’ homes, with the exception of sociodemographics collected during telephone screening. All interviews and test procedures were consistently administered by research staff who had received extensive training in all aspects of assessment. Sociodemographic characteristics included age, gender, martial status, living arrangements, and years of education. Clinical information included number of medications, body mass index (BMI), and nutritional status as assessed by Mini Nutritional Assessment-Short Form [29]. The CFS [22] was used to assess subjective frailty, and the FFP [30] to assess objective frailty based on the five Fried frailty criteria: (1) self-reported unintentional weight loss (> 4.5 kg in the past year); (2) exhaustion (two items from the Center for Epidemiological Studies Depression Scale [31]); (3) low physical activity (< 150 min of moderate-to-vigorous physical activity per week in accordance with the WHO physical activity guidelines [32], measured with the German Physical Activity Questionnaire 50 + [33]); (4) slowness (gender- and height-adjusted slow gait speed), and (5) weakness (gender- and BMI-adjusted low handgrip strength). The number of falls in the last 6 months was recorded, and fear of falling was assessed using the Short Falls Efficacy Scale-International (Short FES-I) [34]. Physical capacity was measured using the Short Physical Performance Battery (SPPB) [35], gait speed and 5-chair stand test (as part of the SPPB), and handgrip strength. Self-reported physical activity was assessed with the German Physical Activity Questionnaire 50+ [33]. The use of an assistive mobility device was recorded. Functioning in activities of daily living (ADL) was measured with the function component (FC) of the Late-Life Function and Disability Instrument (LLFDI), and disability and participation with the disability component (DC) of the short version of the LLFDI [36]. The University of Alabama at Birmingham Life‐Space Assessment (LSA) [37] was used to assess life-space mobility. Cognitive status was assessed using the Short Orientation‐Memory‐Concentration Test [26]. Psychosocial characteristics were assessed for global affect (visual analogue scale) [38], loneliness (UCLA 3‐item loneliness scale [39]), and health-related quality of life (EuroQol‐5‐Dimension 5‐Level, EuroQol visual analogue scale) [40]. Social status was assessed using the 6-item Lubben Social Network Scale (LSNS-6) [41]. Costs for the implementation the GP recruitment strategy (informative letter for study registration by GPs, documents for recruitment and eligibility assessment by study-registered GPs, financial compensation to GPs for referring potential participants) and the direct recruitment strategy (flyers in AOK health magazines, articles in local magazines and newspapers, personalized letters to potentially eligible AOK members) were recorded to determine the cost per participant (= total costs per recruitment strategy divided by the number of participants enrolled by that strategy).

### Statistical analyses

Descriptive data are presented as means and standard deviations, medians and interquartile ranges (IQR), or frequency and percentage. Unpaired *t* tests, Mann–Whitney *U* Test, or Chi-Square tests (with Bonferroni adjusted post-hoc tests) were performed to analyze between-group differences in the participant characteristics according to the recruitment strategy (GP recruitment vs. direct recruitment). Statistical analyses were performed using IBM SPSS Statistics for Windows, version 29.0 (IBM Corp., Armonk, NY, USA).

## Results

**Fig. 1:**
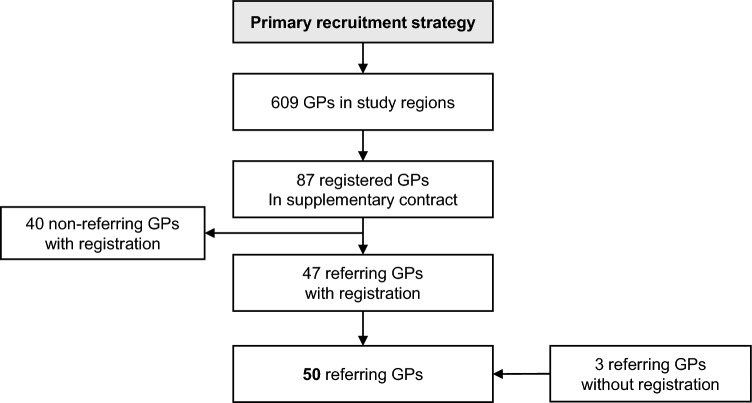
Flowchart of the GP recruitment strategy

Due to the COVID-19 pandemic, the recruitment process was very challenging and only 385 (96.3%) of the initial target of 400 participants could be recruited into the study, despite the implementation of the secondary direct recruitment strategy and the extension of the recruitment period from 12 to 18 months. For the GP recruitment strategy, 87 (14.3%) of the 609 potential GPs at the different study sites signed the supplemental contract and participated in the study. Approximately half of these (*n* = 47, 54.0%) and an additional 3 non-registered GPs referred 95 potential participants to the study after a positive eligibility assessment (Fig. 1).

**Fig. 2:**
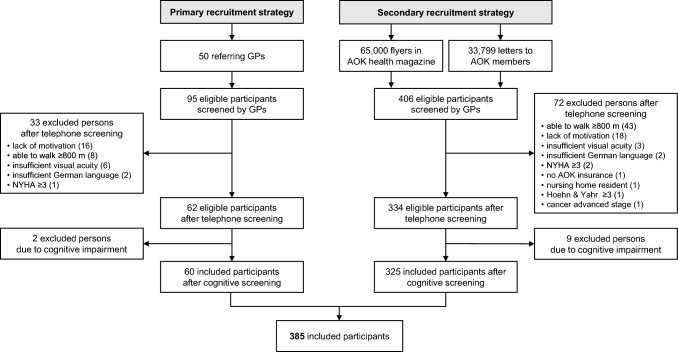
Results of the recruitment strategy illustrated as flow-chart

The direct recruitment strategy yielded 406 potential participants after eligibility assessment by their GPs (Fig. 2). Of the total 501 eligible individuals, 105 (21.0%) were excluded after the telephone screening. The most common reason for exclusion in the direct recruitment was the ability to walk ≥ 800m (59.7%), while participants referred by GPs could mostly not be included due to a lack of motivation to participate after detailed study information was provided (34.7%). At the final cognitive screening at the person’s home, 11 (2.8%) out of the 396 individuals were excluded due to relevant cognitive impairment (SOMCT > 10 pt.), resulting in a final sample size of 385 participants who met all inclusion criteria. A total of 60 (15.6%) participants were enrolled by the GP recruitment strategy and 325 (84.4%) participants were enrolled using direct recruitment strategy.

Table 1 presents the participant characteristics of the total sample and the two subgroups according to GP recruitment and direct recruitment. The mean age of the total sample was 81.2 (SD 5.9) years. Most participants were female (*n* = 283, 73.5%) and lived alone (*n* = 261, 67.8%). The median CFS score was 4 [IQR 4–5] points, indicating pre-frailty based on subjective assessment. More than 85% were classified as pre-frail (*n* = 195, 50.6%) or frail (*n* = 134, 34.8%) according to the FFP. Physical capacity was limited, with a mean SPPB score of 6.5 ± 2.6 points, a mean gait speed of 0.68 ± 0.24 m/s, and a median 5CST duration of 17.7 [IQR 13.1–24.3] s. The mean LLFDI-FC score of 47.6 ± 7.7 points indicated limitations in ADL functioning. Restricted life-space mobility (LSA < 60 pt.) was observed in 72.2% (*n* = 272), more than one-third (*n* = 142, 36.9%) reported a fall history within the last 6 months, and about three-quarters had a moderate (Short FES-I = 7–9 pt.: *n* = 187, 48.6%) or high (Short FES-I ≥ 14 pt.: *n* = 135, 35.1%) fear of falling. Approximately one-sixth were classified as being lonely (UCLA ≥ 6 pt.: *n* = 65, 16.9%), and one-third as being socially isolated (LSNS-6 < 12 pt.: *n* = 128, 33.2%).

**Table 1:** Participants’ characteristics of the total sample and according to GP recruitment and direct recruitment

Variable	Total (*n* = 385)	Recruitment strategy		*p*
		GP (*n* = 60)	Direct (*n* = 325)	
Age, years	81.2 ± 5.9	82.2 ± 5.8	81.1 ± 5.8	0.159
Females, *n*	283 (73.5)	45 (75.0)	238 (73.2)	0.775
Family status, *n*				0.822
Married,	118 (30.6)	17 (28.3)	101 (31.1)	
Married (separated)	4 (1.0)	0 (0.0)	4 (1.2)	
Single	26 (6.8)	3 (5.0)	23 (7.1)	
Divorced	38 (9.9)	6 (10.0)	32 (9.8)	
Widowed	199 (51.7)	34 (56.7)	165 (50.8)	
Education, years	11.3 ± 2.8	10.9 ± 2.7	11.4 ± 2.9	0.239
Living alone, *n*	261 (67.8)	40 (66.7)	221 (68.0)	0.839
Medications	6.8 ± 3.5	7.5 ± 3.3	6.6 ± 3.6	0.095
BMI, kg/m^2^	29.4 ± 5.8	29.5 ± 5.1	29.4 ± 5.9	0.941
MNA-SF, pt	12.7 ± 1.8	12.8 ± 1.8	12.7 ± 1.8	0.716
Nutritional status, *n*				0.672
Normal (MNA-SF ≥ 12 pt.)	310 (80.5)	50 (83.3)	260 (80.0)	
Risk of malnutrition (MNA-SF ≤ 11 pt.)	71 (18.4)	9 (15.0)	62 (19.1)	
Malnutrition (≤ 6 pt.)	4 (1.0)	1 (1.7)	3 (0.9)	
CFS, pt	4 [4, 5]	5 [4, 5]	4 [4, 5]	**0.012**
Fried frailty phenotype^a^, *n*				**0.026**
Robust	56 (14.5)	6 (10.0)	50 (15.4)	
Pre-frail	195 (50.6)	24 (40.0)	171 (52.6)	
Frail	134 (34.8)	30 (50.0)	104 (32.0)	
Fall within last 6 months, *n*	142 (36.9)	18 (30.0)	124 (38.2)	0.229
Short FES-I, pt	11 [9.0–14.0]	10.0 [9.0–16.8]	11.0 [9.0–14.0]	0.402
Fear of falling, *n*				0.735
Low (Short FES-I = 7–9 pt.)	63 (16.4)	8 (13.3)	55 (16.9)	
Moderate (Short FES-I = 10–13 pt.)	187 (48.6)	29 (48.3)	158 (48.6)	
High (Short FES-I ≥ 14 pt.)	135 (35.1)	23 (38.3)	112 (34.5)	
SPPB, pt	6.5 ± 2.6	5.8 ± 2.9	6.7 ± 2.6	**0.021**
Gait speed, m/s	0.68 ± 0.24	0.64 ± 0.26	0.68 ± 0.23	0.255
5CST, s (*n* = 326)	17.7 [13.1–24.3]	18.3 [13.5–27.7]	17.6 [13.0–23.3]	0.338
Handgrip strength, kg	21.8 ± 8.9	22.1 ± 10.8	21.8 ± 8.5	0.841
GPAQ-50 + , METh/week	66.0 [42.3–93.9]	58.4 [40.8–111.4]	66.1 [43.1–91.5]	0.151
Assistive mobility device, *n*	273 (29.1)	45 (75.0)	228 (70.2)	0.448
LLFDI function component, pt	47.6 ± 7.7	45.9 ± 8.0	47.9 ± 7.6	0.083
SF-LLFDI disability component, pt				
Frequency	25.2 ± 4.4	23.6 ± 5.1	25.5 ± 4.2	**0.002**
Limitation	29.5 ± 7.0	27.7 ± 8.3	29.9 ± 6.7	0.063
LSA, pt	49.1 ± 18.7	43.2 ± 21.6	50.1 ± 18.0	**0.023**
Restricted LSM (LSA < 60 pt.), *n*	278 (72.2)	44 (73.3)	234 (72.0)	0.832
Global affect, pt., mean ± SD	67.7 ± 20.1	65.3 ± 20.6	68.1 ± 20.0	0.325
UCLA 3-Item Loneliness Scale, pt	3 [1–4]	3.0 [1.3–4.0]	3.0 [1.0–4.0]	0.638
Lonely (UCLA ≥ 6 pt.), *n*	65 (16.9)	11 (18.6)	54 (16.6)	0.702
SOMCT, pt	4.6 ± 3.0	4.6 ± 2.8	4.6 ± 3.1	0.908
EQ-5D-5L index, pt	0.74 ± 0.22	0.71 ± 0.27	0.75 ± 0.21	0.289
EQ-5D-5L VAS, pt	59.5 ± 18.0	57.2 ± 16.7	59.9 ± 18.3	0.281
LSNS-6, pt	13.9 ± 5.2	13.5 ± 5.7	13.9 ± 5.2	0.562
Social isolation (LSNS-6 < 12 pt.), *n*	128 (33.2)	25 (42.4)	103 (31.7)	0.109

*CFS* Clinical Frailty Scale, *(SF-)LLFDI* (Short Form) Late Life Function and Disability Instrument, *LSA* Life-Space Assessment, *LSM* Life-Space Mobility, *BMI* body mass index, *VAS* visual analogue scale, *Short FES-I* Short Falls Efficacy Scale-International, *FoF* fear of falling, *GPAQ-50+*  German Physical Activity Questionnaire 50+, *LSNS-6* 6-item Lubben Social Network Scale, *MNA-SF* Mini Nutritional Assessment-Short Form, *SPPB* Short Physical Performance Battery, *5CST* 5-chair stand test, *SOMCT* Short Orientation-Memory-Concentration Test

*Data given as mean ± standard deviation, median [interquartile range], or *n* (%). *p* values given for the comparison of GP recruitment and direct recruitment via unpaired *t* tests, Mann–Whitney *U* Test, or Chi-square test

^a^Frailty status categorized according to Fried Frailty Phenotype (robust < 1 pt., pre-frail 1–2 pt., frail ≥ 3 pt.)

Significant differences in participant characteristics were observed according to recruitment strategy (Table 1). Participants recruited by GPs had significantly higher levels of subjective frailty (median CFS: 5 [IQR 4–5] pt. vs. 4 [IQR 4–5] pt., *p* = 0.012), were more often frail according to the objective FFP (50% vs. 32%, Bonferroni adjusted *p* = 0.043), showed lower physical capacity (mean SPPB: 5.8 ± 2.9 pt. vs. 6.7 ± 2.6 pt., *p* = 0.021), and less frequent participation (mean LLDFI-DC, frequency: 23.6 ± 5.1 pt. vs. 25.5 ± 4.2 pt., *p* = 0.002), and lower life-space mobility (mean LSA: 43.2 ± 21.6 pt. vs. 50.1 ± 18.0 pt., *p* = 0.023) as compared to those enrolled via the direct recruitment strategy. Post-hoc power analysis revealed that these significant differences (*p* < 0.05) were detected with a statistical power of 64% for the SPPB, 68% for the FFP, 75% for the LSA, 80% for the CFS, and 87% for the LLDFI-DC.

The total costs for the GP recruitment strategy were €5659 (financial compensation for referrals = €3780; informative letters = €1309, documents for study-registered GPs = €570), while those for the direct recruitment strategy were €69,280 (personalized letters = €53,678, advertising articles = €10,576, flyer in AOK health magazines = €5026). Consequently, costs per randomized participants were €94 for the GP recruitment strategy and €213 for the direct recruitment strategy.

## Discussion

The results of this analysis showed that the different recruitment strategies had an impact on the characteristics of the participants. Participants enrolled through GP recruitment were more frail and showed lower physical capacity, participation, and life-space mobility as compared to those enrolled through direct recruitment (flyers, newspaper articles, personalized letters).

Despite taking into account key aspects for successful recruitment and enrollment of (pre-)frail older adults in clinical trials (i.e. continuous monitoring/adaption of the recruitment strategy, GP involvement, direct communication) [18, 21] and extending the recruitment period from the initial 12 months to 18 months, the targeted sample size of the PromeTheus study could not be achieved. On the GP recruitment side, this is probably mainly due to the COVID-19 pandemic and the limited capacity of the GPs during this period. Despite the financial incentive for GPs, only 8% (50 out of 609) of GPs in the study regions referred potential participants. The low recruitment capacity of GPs may be reflected in the fact that only about half (54%) of the GPs registered to participate in the study participation referred potential participants.

The direct recruitment strategy was much more successful, as documented by the more than fivefold higher enrollment rate achieved with this strategy compared to the GP recruitment (84.4% vs. 16.6%). The direct recruitment strategy involved mailing 33,799 personalized letters to AOK member potentially eligible for participation and distributing 65,000 flyers in the AOK health magazines, which may have resulted in some potential participants being contacted twice. The resulting recruitment rate from these efforts can be estimated to be less than 1% (direct mailings: 325 /33,799 = 0.96%, mailings & flyers: 325/98,799 = 0.33%). The recruitment rates observed here are consistent with those seen in other preventive lifestyle interventions targeting older adults. For instance, the SHAPE project utilized direct mailing of brochures and cover letters to 1477 households to recruit inactive older adults for a neighborhood walking trial, achieving a recruitment rate under 1% with 39 participants enrolled [42]. A potential more effective approach regarding the direct mailings was used in the “Lifestyle Matters RCT” and the “Putting Life in Years RCT” having GPs send personalized invitation letters, study leaflets, and pre-paid response cards to potential participants. These trials including the GPs in the mailings have achieved a 2.3% recruitment rate [43]. The cost of recruiting and enrolling a participant in the PromeTheus study was about half as expensive via the GP recruitment (€94) as via the direct recruitment strategy (€213). These results align with findings from the “Community Aging in Place, Advancing Better Living for Elders-trial”, which identified direct mailing as the most effective yet most expensive recruitment method per participant [44]. To accurately assess the cost-effectiveness of recruitment strategies, whether they involve medical referrals or direct mailings, it is essential to standardize the analysis and reporting methods [45].

The overall participant characteristics of the study sample are comparable to the recent prevention trials in community-dwelling (pre-)frail older people, such as the Australian FIT trial [7] or the Finnish HIPFRA (Home physiotherapy for HIP fracture and FRAilty) trial [46]. The differences in participant characteristics with respect to the recruitment strategy, showing that participants recruited through GPs were more frail and less physically fit, participative, and mobile in their life space, may be due to an effect of extrinsic and intrinsic motivation. The most common reason for an exclusion after GP recruitment was “lack of motivation” (*n* = 16, 34.7%). This could be explained by a primary extrinsic motivation following the recommendation of the GP in his role as a confidant. After the GP referral, these individuals decided not to participate after receiving and discussing detailed information about the study procedure with the study staff. On the other hand, the main reason for exclusion after the direct recruitment strategy was the ability to walk ≥ 800m (*n* = 43, 59.7%). This suggests a higher intrinsic motivation of physically more fit individuals to participate in programs with physical exercise as a main component, also reported as the “healthy volunteer” effect [47]. This aspect may also explain the differences in the lower levels of frailty and higher physical capacity, participation, and life-space mobility in participants recruited directly.

However, the finding of higher limitations among participants recruited through the GP, also illustrates that those with a higher need for support and prevention measures are likely to be better reached through via a recruitment strategy involving health professionals such as GPs. Sufficient recruitment capacity of GPs seems to be crucial in this context. Following the multidisciplinary geriatric perspective, other health professionals such as physiotherapists, occupational therapists, speech therapists, or nurses who recognize the need for support and preventive measures could be empowered in the future to support the recruitment of community-dwelling (pre-)frail older people into preventive programs.

This study has some limitations. First, this is a secondary analysis of the PromeTheus study, thus the sample size was not specifically tailored to the research question in this study and there was a risk of the analyses being underpowered. Second, the study was conducted during the COVID-19 pandemic, which may have had a major impact not only on the recruitment and referral of potential participants by the GPs, but also the recruitment rates of vulnerable, (pre-)frail older adults into research studies in general during this period. Finally, the calculated costs for the recruitment strategies only referred to recruitment and enrollment-related materials (e.g., flyers, newspaper articles, personalized letters, advertising articles, GP documents) and the GP compensation. Staff salary and time were not included, so the recruitment costs were not comprehensive.

## Conclusion

Different recruitment strategies may be required to successfully enroll (pre-)frail home-living older persons in prevention programs. Direct recruitment strategies, in which potential participants are informed directly about the prevention program, appear to be more promising than GP recruitment but may result in enrolment of persons with less functional impairment and higher costs.

## Data Availability

The data sets used and/or analyzed during the current study are available from the corresponding author upon reasonable request.
