# Correction: Prevention at home in older persons with (pre-)frailty: analysis of participants’ recruitment and characteristics of the randomized controlled PromeTheus trial

**DOI:** 10.1007/s40520-024-02837-0

**Published:** 2024-09-23

**Authors:** Tim Fleiner, Corinna Nerz, Michael Denkinger, Jürgen M. Bauer, Christian Grüneberg, Judith Dams, Martina Schäufele, Gisela Büchele, PromeTheus Study Group, Kilian Rapp, Christian Werner

**Affiliations:** 1 Institute for Geriatric Research, Ulm University Medical Center, Ulm, Germany; 2 Geriatric Center, Agaplesion Bethesda Clinic, Ulm, Germany; 3 Department of Clinical Gerontology, Robert-Bosch-Hospital, Stuttgart, Germany; 4 Geriatric Center, Heidelberg University Hospital, Agaplesion Bethanien Hospital Heidelberg, Heidelberg, Germany; 5 Department of Applied Health Sciences, Hochschule für Gesundheit (University of Applied Sciences), Bochum, Germany; 6 Department of Health Economics and Health Services Research, University Medical Centre Hamburg-Eppendorf, Hamburg, Germany; 7 Department of Social Work, University of Applied Sciences, Mannheim, Germany; 8 Institute of Epidemiology and Medical Biometry, Ulm University, Ulm, Germany

**Correction: Aging Clinical and Experimental Research (2024) 36:120** 10.1007/s40520-024-02775-x

In this article Kilian Rapp and Christian Werner were deleted by the Springer production team after proof correction stage.

Members of the PromeTheus group have also been deleted and were not listed in the Acknowledgements. Members of the PromeTheus study group: Jürgen M. Bauer, Christian Werner, Bastian Abel, Natalie Hezel, Michael Denkinger, Nacera Wolf-Belala, Dhayana Dallmeier, Vanessa Haug, Tim Fleiner, Kilian Rapp, Corinna Nerz, Christoph Endress, Rebekka Leonhardt, Erkin Uysal, Monika Dudek, Clemens Becker, Christian Grüneberg, Christian Thiel, Tobias Braun, Rainer Muche, Gisela Büchele, Dietrich Rothenbacher, Birgit Och, Sarah Enderle, Martin Rehm, Martina Schäufele, Ingrid Hendlmeier, Hans-Helmut König, Judith Dams, Sophie Gottschalk, Simone Deininger, Rüdiger Kucher, Anna Lena Flagmeier, Maria Gonzales Medina.

The original article has been corrected.

